# Assessment of the individual and compounding effects of marginalization factors on injury severity, discharge location, recovery, and employment outcomes at 1 year after traumatic brain injury

**DOI:** 10.3389/fneur.2022.942001

**Published:** 2022-08-26

**Authors:** Olga Garduño-Ortega, Huihui Li, Michelle Smith, Lanqiu Yao, Judith Wilson, Alejandro Zarate, Tamara Bushnik

**Affiliations:** ^1^Research Department, Rusk Rehabilitation, Grossman School of Medicine, NYU Langone Health, New York, NY, United States; ^2^Department of Population Health, Grossman School of Medicine, NYU Langone Health, New York, NY, United States; ^3^Occupational Therapy Department, Bellevue Hospital, Health and Hospitals, New York, NY, United States

**Keywords:** traumatic brain injury, marginalization, recovery, employment, injury severity, severe TBI, discharge location

## Abstract

**Objective:**

The aim of this study is to examine the effect of eight distinct marginalized group memberships and explore their compounding effect on injury severity, recovery, discharge location, and employment outcomes 1-year after traumatic brain injury (TBI).

**Methods:**

Individuals with medically confirmed, complicated mild-severe TBI (N = 300) requiring inpatient rehabilitation care between the ages of 18 and 65 were recruited at two urban (public and private) health systems between 2013 and 2019. Data were collected from self-report and medical record abstraction. Marginalized group membership (MGM) includes racial and ethnic minority status, less than a high school diploma/GED, limited English proficiency, substance abuse, homelessness, psychiatric hospitalizations, psychiatric disorders, and incarceration history. Membership in four or more of these groups signifies high MGM. In addition, these factors were explored individually. Unadjusted and adjusted linear and logistic regressions and Kruskal–Wallis tests were used to assess the associations of interest in RStudio.

**Results:**

After adjusting for age, sex, and cause of injury, compared to TBI patients with low MGM, those with high MGM experience significantly longer post-traumatic amnesia (95% CI = 2.70, 16.50; *p* = 0.007) and are significantly more likely to have a severe TBI (per the Glasgow-Coma Scale) (95% CI = 1.70, 6.10; *p* ≤ 0.001) than a complicated mild-moderate injury. Individuals with high MGM also are significantly less likely to be engaged in competitive paid employment 1 year after injury (95% CI = 2.40, 23.40; *p* = 0.001). Patients with high MGM are less likely to be discharged to the community compared to patients with low MGM, but this association was not significant (95% CI = 0.36, 1.16; *p* = 0.141). However, when assessing MGMs in isolation, certain associations were not significant in unadjusted or adjusted models.

**Conclusion:**

This exploratory study's findings reveal that when four or more marginalization factors intersect, there is a compounding negative association with TBI severity, recovery, and employment outcomes. No significant association was found between high MGM and discharge location. When studied separately, individual MGMs had varying effects. Studying marginalization factors affecting individuals with TBI has critical clinical and social implications. These findings underline the importance of addressing multidimensional factors concurrent with TBI recovery, as the long-term effects of TBI can place additional burdens on individuals and their economic stability.

## Introduction

Individuals who are marginalized or disenfranchised based on “their identities, associations, experiences, and environments” (1) are systematically constrained from economic, sociocultural, and political participation, which pushes them further to the peripheries of society (2). Marginalization is deeply enmeshed within structural and health inequities, as evidence suggests the experience and perception of marginalization are linked to poor health outcomes and limited healthcare access (3). The process of marginalization can also create or exacerbate these inequities, leaving the individual vulnerable to cumulative negative health impacts (2). Intersectionality theory has demonstrated how multiple marginalities combine to create a person's unique disadvantages and, therefore, must be conceptualized as a whole (4). Despite the exponential growth of cumulative effect approaches in social and health disparity research, limited research exists within traumatic brain injury (TBI) populations (5–9).

TBI is a leading cause of disability and death in the US and worldwide (10–12). TBI presents a serious and prevalent public health problem, as it is estimated that about half of the world's population will sustain one in their lifetime (12). Recognized as a chronic condition, TBI can lead to a wide range of physical, behavioral, cognitive, and psychological effects, which negatively affect participation and functioning in educational, vocational, social, and everyday activities (13–16). Combined with increased morbidity and mortality risks, TBI has multilevel negative outcomes that can trigger other conditions and diseases, such as epilepsy, depression, anxiety, and dementia (14, 17–19). These complex chronic effects impose increased psychosocial and economic burden, not only on the individual with the injury but also on their immediate and extended communities (20, 21). Advances in treatment and research have improved traumatic brain injury outcomes; however, health disparities remain across social strata (11, 22, 23).

Disparities in health outcomes have been documented across marginalized groups with TBI (22–24). Individuals with incarceration history have higher rates of TBI and have an increased risk of worse cognitive and psychological effects (25), homelessness, and substance use (24, 26, 27). Individuals with psychiatric conditions have been linked to diminished cognitive functioning 10 years post-TBI (9). While, racial and ethnic minorities present with worse TBI symptoms, recovery, and employment outcomes (15, 28–30), patients with limited English proficiency (LEP) have reported lower levels of social functioning post-TBI (31). Strikingly, disparities among minorities have been documented across the continuum of care, including acute and post-acute care, diagnosis, adjustment, recovery, and long-term outcomes (32). Despite the complexity and heterogeneity in TBI effects in conjunction with marginalization factors, a key shortcoming in TBI clinical treatment protocols usually fails to comprehensively consider differences between patients in their trajectories and outcomes following the injury (12, 33). A holistic understanding of the interaction between TBI and existing or new marginalization and inequities is required to develop an intersectional approach that better informs targeted interventions.

Studying marginalization factors affecting individuals with TBI is a critical step that has clinical and social implications. Current research on TBI outcomes and recovery fails to thoroughly assess the interaction of multidimensional marginalization factors, often focusing on a singular dimension (7, 8). Moreover, previous literature has labeled systemic-related factors as vulnerabilities (7), which “refers to a state of being exposed to and unprotected from health-damaging environment” (2). In this study, we chose to use marginalized group memberships (MGM) to denote historically disenfranchised populations due to systemic and social inequities. There is a limited understanding of how MGM cumulatively and differentially interacts with TBI outcomes and recovery. This exploratory study examines the effect of eight distinct marginalized group memberships and explores their compounding effect on injury severity, recovery, discharge location, and employment outcomes.

## Materials and methods

### Participants and setting

Individuals with medically confirmed, complicated mild-severe TBI (N = 300) between the ages of 18–65 were recruited between March 2013 and June 2019 from inpatient rehabilitation units at two health systems located in the New York Metropolitan area for Rusk Rehabilitation Traumatic Brain Injury Model Systems (RRTBIMS), one of 16 centers of the federally funded TBI Model Systems (TBIMS). These health systems are within private and public hospital settings, serving a uniquely diverse, urban community. An exploratory, secondary analysis was conducted on data collected at the time of injury and 1 year after the injury as part of the TBIMS National Database^1^, a prospective, longitudinal study examining the TBI recovery across functional and psychosocial outcomes, as well as assessments of unique to RRTBIMS identifying homelessness and incarceration history.

The TBIMS inclusion criteria are as follows: (1) TBI diagnosis (Glasgow Coma Scale (GCS) score of ≤ 13 or post-traumatic amnesia (PTA) greater than 24 h or loss of consciousness < 30 min at the time of injury); (2) 16 years or older; (3) admission to a TBIMS-affiliated emergency room within 72 h of injury, followed by inpatient rehabilitation to a TBIMS-affiliated program; and (4) completion of written informed consent by an individual with TBI or their family or legal guardian. Since this study explores employment outcomes, participants above the age of 65 years were excluded from this secondary analysis, instead focusing on individuals below retirement age. Further details about the TBIMS and eligibility criteria can be found on the TBIMS National Database website (34). The institutional review boards at both systems approved these procedures.

### Measures

Data were collected from self-reports, including pre-injury and injury baseline demographics. Medical records were reviewed for injury severity (per the GCS) and length of PTA and abstracted for the TBIMS database. The socio-demographic assessment included questions identifying age, gender, race, ethnicity, education level, employment status, language spoken at home, cause of injury, incarceration, psychiatric disorders and hospitalizations, and homelessness history. Employment status was assessed at 1-year post-injury. This observational study's period is from the time of injury to 1-year post-injury and missing data may affect the sample size across variables of interest.

Binary categorizations were used to denote eight distinct marginalized group memberships: racial and ethnic minority status, less than High School (HS) diploma/GED, limited English proficiency (LEP), substance abuse, homelessness, psychiatric hospitalizations, psychiatric disorders, and incarceration history. Incarceration history was defined by the conviction of a felony or misdemeanor, as well as overnight stays that exposed the individual to a high-risk environment. Homelessness is defined as a history of current homelessness and housing instability. Incarceration and homelessness were assessed at the time of injury and 1-year post-injury, with TBIMS variables, as well as assessments unique to RRTBIMS. Membership in four or more of these groups signifies high MGM, while low required at least one MGM.

In addition, due to the heterogeneity within MGMs, further nominal exploration beyond dichotomous categorization was completed for three of the MGMs. Racial and ethnic minority MGM was explored by comparing white participants to Blacks, Asians, and Hispanics. Given this as a secondary analysis, we used standard variable definitions for TBIMS and were unable to further explore racial and ethnic groups. Less than HS, MGM was split to compare individuals with less than HS education level to individuals with HS education and College or above. Finally, LEP MGM was split into English-speaking, Spanish-speaking, and other languages.

Discharge location was dichotomized as community or facility based on the TBIMS categorical variable assessing residence after discharge from the acute inpatient rehabilitation unit. Individuals who were transferred to nursing/subacute level care, adult home, or a hospital setting (e.g., rehabilitation, acute care, or other) were assigned the facility discharge location.

Employment status as either competitively employed or unemployed was assessed using the TBIMS follow-up variable at 1-year post-injury. Unemployed was defined as those who reported being unemployed, participating in unpaid activities (e.g., volunteer work and homemaker), on unpaid leave, or students (e.g., full-time/part-time status).

Injury severity was diagnosed using the Glasgow Coma Scale (GCS) which assesses the functions of eye-opening, verbal response, and motor response (35). GCS scores range from 3 to 8 (severe), 9 to 12 (moderate), or 13 to 15 (mild). Throughout this study, reference to mild TBI refers to complex or complicated mild TBI requiring acute inpatient rehabilitation care. The GCS assessment provides the level of consciousness and overall severity of the head injury. Low GCS scores at the time of injury, which denote increased injury severity, have been shown as a significant predictor of TBI outcomes (36, 37).

Post-traumatic Amnesia (PTA) days were used as a measure of TBI recovery. PTA is a state of confusion and memory loss that occurs after brain injury that is measured in days. PTA is considered a good predictor of long-term TBI recovery outcomes (38).

### Analysis

Descriptive statistics were performed for sample characterization. There was no statistical correction for missing data, which was treated as missing at random, including from those lost to follow-up. There were 43, 48, 52, 43, 50, and 53 participants who did not provide age, sex, insurance status, race/ethnicity, education level, and cause of injury, respectively. Linear and Logistic regressions and Kruskal–Wallis tests were used to assess the associations of interest in this exploratory analysis. For this analysis, unadjusted and adjusted logistic regressions were conducted to assess the association between the eight MGMs and the outcomes of injury severity, discharge location, and employment status. Unadjusted models were adjusted for age, sex, and cause of TBI covariates. For PTA days, unadjusted tests were conducted with Kruskal–Wallis test and adjusted analyses of PTA days were conducted with a t-test by fitting linear regressions to examine differences between MGMs and PTA days, respectively. We also investigated the compounding effects of MGM, which evaluated the association between the chosen outcomes and high and low MGM. A *p*-value of < 0.050 was considered statistically significant. All statistical analyses were conducted in RStudio (39).

## Results

### Demographics

#### Characteristics at time of injury

A total of 300 individuals who sustained a moderate to severe TBI and received inpatient rehabilitation between March 2013 and June 2019 were characterized (see Table 1). Individuals were primarily male (88.9%), Hispanic (37.4%), White (27.2%), or Black (23.3%), enrolled in Medicaid (43.1%), had high school level education or above (58.2%), and a fall (47.8%) or violence (19.0%)-related injury. Participants had an average age of 42 years (SD = 14). This study's sample is more racially and ethnically diverse compared to TBI populations that received inpatient rehabilitation care in the United States and enrolled in TBIMS (40).

**Table 1:** Sample characteristics for individuals with TBI at the Time of Injury (N = 300).

**Variable**	**No. (%) or mean (SD) of participants**
**Age**	42.11 (14.01)
**Male sex**	224 (88.9%)
**Insurance status**	
Medicaid	107 (43.1%)
Medicare	7 (2.8%)
Worker's Comp/No Fault	39 (15.7%)
Private or Self-paid	36 (14.5%)
Local Assistance	52 (21.0%)
Other	7 (2.8%)
**Race/Ethnicity**	
Hispanic Origin	96 (37.4%)
White	70 (27.2%)
Black	60 (23.3%)
Asian/Pacific Islander	17 (6.6%)
Native American	1 (0.4%)
Other	13 (5.1%)
**Less than high school level education**	107 (41.8%)
**Cause of injury**	
Fall or Impact	118 (47.8%)
Violence-related	47 (19.0%)
Pedestrian	41 (16.6%)
Vehicular	37 (15.0%)
Sports	2 (0.8%)
Other	2 (0.8%)

Frequencies might not be based on a total of 300 participants due to missing data.

A demographic summary by MGM is shown in Table 2. A total of 47 participants had one MGM, 53 had two MGMs, 68 had three MGMs, 40 had four MGMs, 26 had five MGMs, 9 had six MGMs, 4 had seven MGMs, and 2 had eight MGMs. Finally, 51 had no MGMs. Most participants identified the following MGMs: Racial and ethnic minority status (63.67%), substance abuse (42.67%), less than HS level education (35.67%), and LEP (32%).

**Table 2:** Sample demographic characteristics by marginalized group membership (N = 249).

	**Incarceration History** **(N = 53)**	**Psychiatric disorders** **(N = 75)**	**Psychiatric hospitalizations** **(N = 36)**	**Homelessness** **(N = 59)**	**Substance abuse** **(N = 128)**	**Limited English proficiency** **(N = 96)**	**Less than HS diploma/GED** **(N = 107)**	**Racial/ethnic minority** **(N = 191)**
**AGE [mean (SD)]**	38.81 (12.67)	40.35 (12.12)	39.22 (12.48)	45.22 (13.04)	40.81 (13.23)	42.85 (14.03)	41.44 (13.68)	42.34 (14.14)
**Male sex (%)**	49 (92.5)	61 (83.6)	31 (86.1)	55 (93.2)	117 (92.1)	85 (90.4)	96 (90.6)	167 (89.3)
**Race/Ethnicity**								
Hispanic Origin	19 (35.8)	20 (26.7)	14 (38.9)	22 (37.3)	49 (38.3)	64 (66.7)	59 (55.1)	96 (50.3)
White	12 (22.6)	31 (41.3)	8 (22.2)	14 (23.7)	42 (32.8)	15 (15.6)	13 (12.1)	4 (2.1)
Black	18 (34.0)	17 (22.7)	11 (30.6)	20 (33.9)	27 (21.1)	5 (5.2)	27 (25.2)	60 (31.4)
Asian/Pacific Islander	1 (1.9)	2 (2.7)	1 (2.8)	0 (0.0)	4 (3.1)	8 (8.3)	3 (2.8)	17 (8.9)
Native American	1 (1.9)	1 (1.3)	0 (0.0)	1 (1.7)	1 (0.8)	0 (0.0)	1 (0.9)	1 (0.5)
Other	2 (3.8)	4 (5.3)	2 (5.6)	2 (3.4)	5 (3.9)	4 (4.2)	4 (3.7)	13 (6.8)
**Language spoken at home**								
English	39 (73.6)	61 (81.3)	29 (80.6)	42 (71.2)	90 (70.3)	0 (0.0)	52 (48.6)	108 (56.5)
Spanish	10 (18.9)	9 (12.0)	5 (13.9)	13 (22.0)	30 (23.4)	65 (67.7)	45 (42.1)	65 (34.0)
Other	4 (7.5)	5 (6.7)	2 (5.6)	4 (6.8)	8 (6.2)	31 (32.3)	10 (9.3)	18 (9.4)
**Education level**								
Less than High School	26 (49.1)	32 (42.7)	15 (41.7)	37 (64.9)	52 (41.9)	55 (59.8)	107 (100.0)	94 (50.8)
High School or GED	19 (35.8)	20 (26.7)	13 (36.1)	14 (24.6)	36 (29.0)	18 (19.6)	0 (0)	46 (24.9)
Some college or above	8 (15.1)	23 (30.7)	8 (22.2)	6 (10.5)	36 (29.0)	19 (20.7)	0 (0)	45 (24.3)
**Insurance status**								
Medicare	0 (0.0)	3 (4.1)	1 (2.8)	1 (1.7)	5 (4.0)	1 (1.1)	1 (1.0)	5 (2.7)
Medicaid	34 (65.4)	42 (57.5)	26 (72.2)	40 (69.0)	57 (45.6)	34 (37.0)	48 (46.6)	86 (47.0)
Worker's Comp/No Fault	8 (15.4)	5 (6.8)	0 (0.0)	2 (3.4)	14 (11.2)	17 (18.5)	15 (14.6)	32 (17.5)
Private/Self	3 (5.8)	8 (11.0)	3 (8.3)	5 (8.6)	19 (15.2)	9 (9.8)	9 (8.7)	16 (8.7)
Local Assistance	5 (9.6)	12 (16.4)	4 (11.1)	10 (17.2)	27 (21.6)	30 (32.6)	29 (28.2)	40 (21.9)
Other	2 (3.8)	3 (4.1)	2 (5.6)	0 (0.0)	3 (2.4)	1 (1.1)	1 (1.0)	4 (2.2)
**Cause of injury**								
Fall or Impact	23 (46.0)	38 (52.8)	20 (55.6)	32 (58.2)	67 (54.0)	45 (48.9)	50 (49.0)	86 (47.3)
Violence-related	10 (20.0)	18 (25.0)	6 (16.7)	15 (27.3)	26 (21.0)	15 (16.3)	25 (24.5)	34 (18.7)
Pedestrian	7 (14.0)	12 (16.7)	7 (19.4)	6 (10.9)	20 (16.1)	18 (19.6)	10 (9.8)	29 (15.9)
Vehicular	8 (16.0)	3 (4.2)	2 (5.6)	2 (3.6)	10 (8.1)	13 (14.1)	15 (14.7)	29 (15.9)
Sports	1 (2.0)	0 (0.0)	0 (0.0)	0 (0.0)	1 (0.8)	1 (1.1)	1 (1.0)	2 (1.1)
Other	1 (2.0)	1 (1.4)	1 (2.8)	0 (0.0)	0 (0.0)	0 (0.0)	1 (1.0)	2 (1.1)

Frequencies might not be based on a total of 249 participants due to missing data.

#### Compounding effects of marginalized group membership

Among participants with at least one MGM (N = 249), about 33% identified high MGM. Participants with high MGM were primarily male (91.2%), Hispanic (51.9%), Black (27.2%), or White (13.6%), enrolled in Medicaid (64.6%), had less than HS level education (70.4%), and a fall (52.6%), violence (23.7%), or pedestrian (14.5%)-related injury; with an average age of 40.7 years (SD = 12.6). Participants with low MGM were primarily male (88.4%), Hispanic (32.1%), White (30.4%), or Black (22.6%), enrolled in Medicaid (32.3%), Workers Comp or No fault (19.9%) or Private (18.0%) insurance, had more than high school level education (29.9%), and a fall (46.0%) or vehicular (17.8%)-related injury.

Among participants (N = 249) with at least one marginalized group membership, the impact of the level of marginalization was explored (see Table 3). Compared to low MGM, individuals with high MGM had 2.61 times the odds (95% CI = 1.50, 4.70; *p* = 0.001) of sustaining a severe TBI as opposed to a complicated mild-moderate TBI. After adjusting for age, sex, and cause of TBI, the odds of sustaining a severe TBI instead of complicated mild-moderate TBI increased to 3.17 times the odds (95% CI = 1.70, 6.10; *p* ≤ 0.001) for those with high MGM.

**Table 3:** Unadjusted and adjusted regression models predicting injury severity, discharge location, employment, and pta days outcomes for low vs. high marginalized group membership (N = 249).

**Variables**	**High MGM**					
	**Unadjusted**			**Adjusted**		
	**OR (SD)**	**95% CI**	***p*** **-value**	**OR (SD)**	**95% CI**	***p*** **-value**
Injury severity—severe^a^	2.61 (1.35)	(1.50, 4.70)	0.001**	3.17 (1.39)	(1.70, 6.10)	<0.001***
Discharge location—to community	0.63 (1.32)	(0.37, 1.09)	0.097^†^	0.65 (1.34)	(0.36, 1.16)	0.141
Employment—unemployed	5.80 (1.74)	(2.20, 20.10)	0.001**	6.57 (1.77)	(2.40, 23.4)	0.001**
	**Difference (SD)**	**95% CI**	***p*** **-value**	**Difference (SD)**	**95% CI**	***p*** **-value**
PTA days	9.16 (3.37)	(2.50, 15.8)	0.007**	9.58 (3.50)	(2.70, 16.5)	0.007**

Adjusted for age, sex, and cause of TBI. Sample size may vary due to missing data. The reference group are participants with low marginalized group membership.

^†^*p* < 0.10, *p < 0.05, **p < 0.01, ***p < 0.001.

^*a*^Reference level of Injury Severity: complicated mild or moderate injury.

Individuals with high MGM also experienced about 9.16 (95% CI = 2.50, 15.80; *p* = 0.007) more days of PTA, significantly more than low-MGM participants. After adjusting for age, sex, and cause of TBI, the average PTA days are 25.70 (SD = 27.50) for participants with high MGM and 16.90 (SD = 18.20) for low MGM participants, with a significant difference of about 9.58 days (95% CI = 2.70, 16.50; *p* = 0.007).

Compared with the low-marginalized group, high-marginalized participants had 0.63 times the odds, or 37% lower odds, of being discharged to the community (95% CI = 0.37, 1.09; *p* = 0.097). After adjusting for covariates, high-marginalized participants had 0.65 times the odds of being discharged to the community (95% CI = 0.36, 1.16; *p* = 0.141). However, these unadjusted and adjusted associations were not significant.

In relation to employment, participants with high MGM had 5.80 times the odds of being unemployed 1 year after discharge (95% CI = 2.20, 20.10; *p* = 0.001). In adjusted models, the odds of unemployment increased to 6.57 the odds (95% CI = 2.40, 23.4; *p* = 0.001) for participants with high MGM. Overall, individuals with high MGM had worse outcomes with respect to injury severity, PTA, and employment regardless of the combination of MGMs.

#### Individual effects of marginalized groups and outcomes

**Figure 1:**
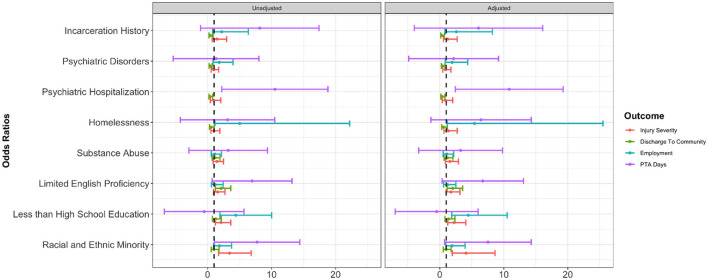
Individual marginalization factors effects and confidence intervals from unadjusted and adjusted regression models across injury severity, discharge location, employment and Post-Traumatic Amnesia (PTA) days. Unadjusted and adjusted logistic regressions were conducted for each MGM and outcome. The odds ratios and the corresponding confidence intervals were provided as horizontal bars. The right panel shows the regression results with adjustment of age, sex, and cause of TBI. The vertical line in each panel marks where the value equals 1. The y-axis provides eight individual MGMs. The x-axis shows the values that represent the odds ratios or differences in mean. For PTA days, Kruskal–Wallis test and linear regressions adjusting for age, sex, and cause of TBI were conducted. The differences in PTA days and their confidence intervals are shown. The results of psychiatric hospitalization and employment were omitted due to the large range, as shown in **Table 9**.

The individual effects of each MGM were explored across injury severity, discharge location, employment, and PTA days (see Figure 1). Findings reveal that when studying MGMs in isolation, certain associations were not significant in unadjusted or adjusted models.

### Racial and ethnic minority status MGM

Among 257 participants, racial and ethnic minority outcomes were explored (see Table 4). Compared to whites, racial and ethnic minority participants had 3.43 times the odds (95% CI = 1.8, 7.0; *p* ≤ 0.001) of sustaining a severe TBI as opposed to a complicated mild-moderate TBI; after adjusting for age, sex, and cause of TBI, minority participants' odds increased to 4.11 (95% CI = 2.0, 8.9; *p* ≤ 0.001) of sustaining a severe TBI. Differences among Blacks, Asians, and Hispanics were found in injury severity when compared to whites. The odds ratios of sustaining a severe TBI as opposed to a complicated mild-moderate TBI were 3.06 (95% CI = 1.4, 7.0; *p* = 0.007) for Blacks and 3.74 (95% CI = 1.9, 7.8; *p* < 0.001) for Hispanics. The adjusted odds ratios of sustaining a severe TBI as opposed to a complicated mild-moderate TBI increased for both Blacks at 3.68 (95% CI = 1.52, 9.24; *p* = 0.004) and for Hispanics at 4.42 (95% CI = 2.06, 9.93; *p* < 0.001) compared to whites. Asians had 3.68 times the odds (95% CI = 0.92, 12.48; *p* = 0.061) of sustaining a severe TBI after adjusting for covariates, but this association was not significant.

**Table 4:** Unadjusted and adjusted regression models predicting injury severity, discharge location, employment, and PTA days outcomes for Racial and Ethnic Minority Marginalized Group Membership (N = 257).

	**Unadjusted**			**Adjusted**		
**Variable**	**OR (SD)**	**95% CI**	***p*-value**	**OR (SD)**	**95% CI**	***p*-value**
Injury severity—severe^a^	3.43 (1.42)	(1.8, 7.0)	<0.001***	4.11 (1.46)	(2.0, 8.9)	<0.001***
Black	3.06 (1.52)	(1.4, 7.0)	0.007**	3.68 (1.58)	(1.52, 9.24)	0.004**
Asian	2.00 (1.81)	(0.6, 6.3)	0.244	3.43 (1.93)	(0.92, 12.48)	0.061^†^
Hispanic	3.74 (1.44)	(1.9, 7.8)	<0.001***	4.42 (1.49)	(2.06, 9.93)	<0.001***
Discharge location—to community	0.99 (1.35)	(0.55, 1.77)	0.986	0.99 (1.35)	(0.54, 1.78)	0.960
Black	0.67 (1.44)	(0.33, 1.38)	0.283	0.64 (1.47)	(0.30, 1.36)	0.241
Asian	0.86 (1.74)	(0.29, 2.64)	0.791	0.82 (1.76)	(0.27, 2.56)	0.721
Hispanic	1.25 (1.39)	(0.65, 2.39)	0.503	1.23 (1.4)	(0.63, 2.41)	0.536
Employment - Unemployed	1.83 (1.44)	(0.89, 3.72)	0.095^†^	1.91 (1.45)	(0.91, 3.98)	0.084^†^
Black	3.94 (1.69)	(1.48, 11.83)	0.009**	5.47 (1.81)	(1.83, 19.35)	0.004**
Asian	0.73 (1.95)	(0.19, 2.82)	0.634	0.53 (2.06)	(0.13, 2.25)	0.380
Hispanic	2.47 (1.52)	(1.10, 5.73)	0.031*	2.55 (1.55)	(1.09, 6.15)	0.032*
	**Difference (SD)**	**95% CI**	***p*** **-value**	**Difference (SD)**	**95% CI**	***p*** **-value**
PTA Days	7.72 (3.41)	(1–14)	0.025*	7.52 (3.45)	(0.7, 14.3)	0.031*
Black	8.38 (3.85)	(0.78, 15.97)	0.031*	7.70 (4.01)	(−0.22, 15.61)	0.057^†^
Asian	0.12 (5.81)	(−11.33, 11.58)	0.983	1.42 (5.98)	(−10.39, 13.23)	0.812
Hispanic	8.49 (3.36)	(1.86, 15.12)	0.012*	8.29 (3.44)	(1.51, 15.08)	0.017*

Adjusted for age, sex, and cause of TBI. Sample size may vary due to missing data. The variable “Racial and Ethnic Minority” was treated as both binary and nominal. The reference group for both binary and nominal odds ratios are participants who are white.

^†^*p* < 0.10, *p < 0.05, **p < 0.01, ***p < 0.001.

^*a*^Reference level of Injury Severity: complicated mild or moderate injury.

The average PTA days are 21.28 (SD = 21.63) for racial and ethnic minority participants and 13.56 (SD = 20.33) for white participants, with a significant difference of about 7.72 days (*p* = 0.025). In adjusted models, minorities experienced 7.52 (95% CI = 0.70, 14.30; *p* = 0.031) more PTA days significantly more than white participants. Before adjusting for age, sex, and cause of TBI, Black participants, in particular, had 8.38 (95% CI = 0.78, 15.97; *p* = 0.031) more PTA days, while Hispanics presented 8.49 (95% CI = 1.86, 15.12; *p* = 0.012) more PTA days. After adjusting, Hispanics had 8.29 (95% CI = 1.51, 15.08; *p* = 0.017) more PTA days compared to whites; furthermore, Blacks had an average of 7.7 more PTA days, but this association was marginally significant (95% CI = −0.22, 15.61; *p* = 0.057).

Employment outcomes at 1-year post-injury also revealed significant differences between racial and ethnic minorities and white participants. Blacks, for instance, had 3.94 times the odds (95% CI = 1.48, 11.83; *p* = 0.009), while Hispanics had 2.47 times the odds (95% CI = 1.10, 5.73; *p* = 0.031) of being unemployed 1 year after discharge compared to whites. The adjusted unemployment odds are 5.47 times (95% CI = 1.83, 19.35; *p* = 0.004) for Blacks and 2.55 (95% CI = 1.09, 6.15; *p* = 0.032) for Hispanics. Despite these outcomes, differences between racial and ethnic minorities and whites were not significant in unadjusted (*p* = 0.095) or adjusted models (*p* = 0.084). No significant association was found between racial and ethnic minority status MGM and discharge location. Furthermore, no significant associations were found for Asians across all outcome variables, which may be attributed to the limited sample size (N = 17) of Asians in this study.

### Less than high school education MGM

Among 256 participants, educational attainment's effect on outcomes of interest was explored as shown in Table 5. Participants with less than HS education had 2.11 times the odds of sustaining a severe TBI, as opposed to a complicated mild-moderate injury, when compared to people with a High School education level or above (95% CI = 1.20, 3.60; *p* = 0.007). After adjusting for age, sex, and cause of TBI, people with less than HS education had 2.25 times the odds of sustaining a severe TBI as compared to people with HS education level or above (95% CI = 1.20, 4.10; *p* = 0.008). Differences were found among education levels and injury severity. People with high school education had 0.48 times the odds, or 52% lower odds (95% CI = 0.24,0.95; *p* = 0.037), and people with college-level education had 0.44 times the odds or 56% lower odds (95% CI = 0.23,0.83; *p* = 0.013) of sustaining a severe TBI when compared to individuals with less than HS education. The adjusted odds ratios of sustaining a severe TBI as opposed to a complicated mild-moderate TBI were 0.40 for individuals with HS education (95% CI = 0.18, 0.83; *p* = 0.016) and 0.45 for individuals with college-level education (95% CI = 0.22, 0.90; *p* = 0.025).

**Table 5:** Unadjusted and adjusted regression models predicting injury severity, discharge to community, employment, and PTA days outcomes for Less than High School Education Marginalized Group Membership (N = 256).

	**Unadjusted**			**Adjusted**		
**Variables**	**OR (SD)**	**95% CI**	***p*-value**	**OR (SD)**	**95% CI**	***p*-value**
Injury severity—severe^a^	2.11 (1.32)	(1.20, 3.60)	0.007**	2.25 (1.36)	(1.20, 4.10)	0.008**
HS diploma	0.48 (1.42)	(0.24, 0.95)	0.037*	0.40 (1.47)	(0.18, 0.83)	0.016**
College or above	0.44 (1.39)	(0.23, 0.83)	0.013*	0.45 (1.43)	(0.22, 0.90)	0.025*
Discharge location—to community	1.26 (1.30)	(0.75, 2.13)	0.390	1.37 (1.32)	(0.79, 2.79)	0.262
HS diploma	0.63 (1.39)	(0.33, 1.19)	0.155	0.55 (1.41)	(0.28, 1.07)	0.080
College or above	0.85 (1.37)	(0.46, 1.56)	0.593	0.82 (1.39)	(0.43, 1.56)	0.546
Employment—unemployed	4.39 (1.43)	(2.00, 11.00)	<0.001***	4.30 (1.55)	(2.00, 11.00)	0.001**
HS diploma	0.39 (1.66)	(0.14, 1.06)	0.066^†^	0.43 (1.69)	(0.15, 1.20)	0.107
College or above	0.15 (1.57)	(0.06, 0.35)	<0.001***	0.13 (1.62)	(0.05, 0.32)	<0.001***
	**Difference (SD)**	**95% CI**	***p*** **-value**	**Difference (SD)**	**95% CI**	***p*** **-value**
PTA days	−0.54 (3.18)	(−6.8, 5.8)	0.867	−0.50 (3.30)	(−7.0, 6.0)	0.879
HS diploma	2.74 (3.52)	(−4.2, 9.7)	0.438	2.25 (3.64)	(−4.9, 9.4)	0.537
College or above	−3.07 (3.36)	(−9.7, 3.6)	0.362	−2.76 (3.48)	(−9.6, 4.1)	0.429

Adjusted for age, sex, and cause of TBI. Sample size may vary due to missing data. The variable “Less than High school Education” was treated as both binary and nominal. The reference group for binary odds ratios are participants that have “High School education and above.” The reference group for nominal odds ratios are participants with “Less than High School education.”

^†^*p* < 0.10, *p < 0.05, **p < 0.01, ***p < 0.001.

^*a*^Reference level of Injury Severity: complicated mild or moderate injury.

Employment at 1-year post-injury revealed significant differences between education levels. People with less than an HS education level had 4.39 times the odds of being unemployed after discharge as compared to people with an HS education level or above (95% CI = 2.00, 11.00; *p* ≤ 0.001). After adjusting for covariates, participants with less than HS education level had 4.30 times the odds of being employed 1 year after the injury as compared to people with higher education (95% CI = 2.00, 11.00; *p* = 0.001). When exploring the effect of education levels on unemployment, people with college education level or above had 0.15 times the odds, or 85% lower odds, of being unemployed (95% CI = 0.06, 0.35; *p* ≤ 0.001). This finding of college-educated individuals having lower odds of unemployment increased to 87% lower odds in adjusted models (95% CI = 0.05, 0.32; *p* ≤ 0.001). For individuals with HS level education, the odds are 0.39 (95% CI = 0.14, 1.06; *p* = 0.066) in unadjusted models and 0.43 (95% CI = 0.15, 1.20; *p* = 0.107) in adjusted models, but no significant associations were found. Furthermore, no significant associations were found between discharge location, PTA days, and education level.

### LEP MGM

Among 257 participants, LEP's effect on outcomes was explored in adjusted and unadjusted regression models (see Table 6). After adjusting for age, sex, and the cause of TBI, people with LEP had 1.75 times the odds of sustaining a severe TBI as compared to people without LEP (95% CI = 0.98, 3.18), but this association was not significant (*p* = 0.061).

**Table 6:** Unadjusted and adjusted regression models predicting injury severity, discharge location, employment, and PTA days outcomes for limited English Proficiency Marginalized Group Membership (N = 257).

**Variables**	**Unadjusted**			**Adjusted**		
	**Odds (SD)**	**95% CI**	***p*-value**	**Odds (SD)**	**95% CI**	***p*-value**
Injury severity—severe^a^	1.58 (1.32)	(0.92, 2.72)	0.100	1.75 (1.35)	(0.98, 3.18)	0.061^†^
Spanish	1.61 (1.37)	(0.87, 2.99)	0.126	1.70 (1.40)	(0.87, 3.31)	0.118
Other language	1.50 (1.52)	(0.65, 3.40)	0.335	1.89 (1.58)	(0.77, 4.64)	0.163
Discharge location—to community	2.10 (1.33)	(1.20, 3.70)	0.009**	2.02 (1.34)	(1.10, 3.60)	0.015*
Spanish	2.44 (1.39)	(1.30, 4.78)	0.007**	2.35 (1.4)	(1.23, 4.67)	0.012*
Other language	1.55 (1.53)	(0.69, 3.66)	0.302	1.48 (1.54)	(0.64, 3.59)	0.364
Employment—unemployed	1.20 (1.43)	(0.60, 2.50)	0.615	1.21 (1.45)	(0.59, 2.58)	0.604
Spanish	1.17 (1.52)	(0.53, 2.75)	0.700	1.19 (1.54)	(0.53, 2.88)	0.683
Other language	1.25 (1.73)	(0.45, 4.03)	0.687	1.26 (1.77)	(0.43, 4.25)	0.686
	**Difference (SD)**	**95% CI**	***p*** **-value**	**Difference (SD)**	**95% CI**	***p*** **-value**
PTA days	6.95 (3.18)	(0.67, 13.23)	0.030*	6.7 (3.24)	(0.31, 13.09)	0.040*
Spanish	4.67 (3.61)	(−2.50, 11.8)	0.198	4.35 (3.67)	(−2.90, 11.60)	0.238
Other language	11.92 (4.92)	(2.20, 21.6)	0.016*	11.85 (5.00)	(2.00, 21.70)	0.019*

Adjusted for age, sex, and cause of TBI. Sample size may vary due to missing data. The variable “Limited English Proficiency (LEP)” was treated as both binary and nominal. The reference group for binary odds ratios are participants that have “non-LEP.” The nominal odds ratios are participants are “English-speaking.”

^†^*p* < 0.10, *p < 0.05, **p < 0.01, ***p < 0.001.

^*a*^Reference level of Injury Severity: complicated mild or moderate injury.

The average PTA days are 23.57 (SD = 25.74) for people with LEP and 16.62 (SD = 18.35) for people without LEP, with a significant difference of around 6.95 days (*p* = 0.030). In adjusted models, people with LEP had 6.70 (SD = 3.24, *p* = 0.040) more PTA days compared to people without LEP. Individuals who spoke a language other than English or Spanish presented 11.92 (95% CI = 2.2, 21.6; *p* = 0.016) more PTA days in unadjusted models and 11.85 (95% CI = 2.0, 21.7; *p* = 0.019) more days in adjusted models.

People with LEP had 2.10 times the odds of being discharged to the community as compared to people with English proficiency (95% CI = 1.20, 3.70; *p* = 0.009). After adjusting for covariates, individuals with LEP had 2.02 times the odds of being discharged to the community as compared to those with English proficiency (95% CI = 1.1, 3.6; *p* = 0.015). Spanish-speaking participants, in particular, had 2.44 times the odds of being discharged to the community setting as compared to English speakers (95% CI = 1.30, 4.78; *p* = 0.007). In adjusted models, Spanish-speaking participants presented 2.35 times the odds of discharge to a community setting compared to English-speakers (95% CI = 1.23, 4.67; *p* = 0.012). No significant association was found between LEP and employment status or injury severity.

### Substance abuse MGM

The effects of substance abuse on TBI outcomes were explored as shown in Table 7. Despite participants with substance abuse MGM having higher odds of being in the worse category with respect to TBI severity, discharge location, and employment status, these associations were not significant in either adjusted or unadjusted models. No significant differences were found in PTA days.

**Table 7:** Unadjusted and adjusted regression models predicting injury severity, discharge location, employment, and PTA days outcomes for substance abuse marginalized group membership (N = 256).

**Variable**	**Unadjusted**			**Adjusted**		
	**OR (SD)**	**95% CI**	***p*-value**	**OR (SD)**	**95% CI**	***p*-value**
Injury severity—severe^a^	1.46 (1.31)	(0.86, 2.51)	0.164	1.58 (1.36)	(0.87, 2.91)	0.137
Discharge location—to community	1.16 (1.30)	(0.69, 1.95)	0.573	1.19 (1.33)	(0.68, 2.09)	0.540
Employment—unemployed	1.10 (1.40)	(0.57, 2.14)	0.77	1.07 (1.44)	(0.52, 2.19)	0.846
	**Difference (SD)**	**95% CI**	***p*** **-value**	**Difference (SD)**	**95% CI**	***p*** **-value**
PTA days	3.20 (3.13)	(−3.00, 9.4)	0.307	3.24 (3.34)	(−3.30, 9.80)	0.333

Adjusted for age, sex, and cause of TBI. Sample size may vary due to missing data. The reference group for all odds ratios are the participants who reported no substance abuse.

^†^*p* < 0.10, *p < 0.05, **p < 0.01, ***p < 0.001.

^*a*^Reference level of Injury Severity: complicated mild or moderate injury.

### Homelessness MGM

As depicted in Table 8, people who were homeless had 0.55 times the odds, or 45% lower odds, of being discharged to the community as compared to people who were not homeless (95% CI = 0.29, 1.02), but this association only reached borderline statistical significance (*p* = 0.059). After adjusting for age, sex, and the cause of TBI, this association was not significant (*p* = 0.133).

**Table 8:** Unadjusted and adjusted logistic regression models predicting injury severity, discharge location, employment, and PTA days outcomes for Homelessness Marginalized Group Membership (N = 196).

**Variable**	**Unadjusted**			**Adjusted**		
	**OR (SD)**	**95% CI**	***p*-value**	**OR (SD)**	**95% CI**	***p-*value**
Injury severity—severe^a^	0.99 (1.39)	(0.52, 1.89)	0.982	1.33 (1.45)	(0.64, 2.75)	0.445
Discharge location—To community	0.55 (1.38)	(0.29, 1.02)	0.059^†^	0.59 (1.41)	(0.30, 1.20)	0.133
Employment—unemployed	5.00 (2.15)	(1.40, 32.2)	0.035*	5.40 (2.20)	(1.40, 36.0)	0.032*
	**Difference (SD)**	**95% CI**	***p*** **-value**	**Difference (SD)**	**95% CI**	***p*** **-value**
PTA days	3.10 (3.76)	(−4.30, 10.50)	0.411	6.44 (4.00)	(−1.50, 14.40)	0.110

Adjusted for age, sex, and cause of TBI. Sample size may vary due to missing data. The reference group for all odds ratios are the participants who reported no homelessness.

^†^*p* < 0.10, *p < 0.05, **p < 0.01, ***p < 0.001.

^*a*^Reference level of Injury Severity: complicated mild or moderate injury.

Individuals who were homeless had 5.00 times the odds of being unemployed 1 year post-injury as compared to people who were not homeless (95% CI = 1.40, 32.20; *p* = 0.035). The adjusted unemployment odds for people who were homeless increased to 5.40 times the odds (95% CI = 1.40, 36.0; *p* = 0.032). No significant associations were found between injury severity or PTA days and homelessness.

### Psychiatric hospitalizations MGM

Participants with psychiatric hospitalization history had longer PTA days (M = 28.07; SD = 27.57) than people without psychiatric hospitalization history (M = 17.56; SD = 19.96); the difference is around 10.51 days (*p* = 0.014). After adjusting for age, sex, and the cause of TBI, individuals with psychiatric hospitalizations experienced 10.84 (95% CI = 2.4, 19.3; *p* = 0.013) more PTA days.

People with psychiatric hospitalization had 0.39 times the odds, or 61% lower odds, of being discharged to the community as compared to people without psychiatric hospitalizations (95% CI = 0.18, 0.79; *p* = 0.010). In adjusted models, participants with psychiatric hospitalization had 0.38 times the odds, or 62% lower odds, of being discharged to community settings as compared to people without psychiatric hospitalizations (95% CI = 0.17, 0.80; *p* = 0.011).

In addition, people with psychiatric hospitalizations had 10.91 times the odds of being unemployed as compared to people without psychiatric hospitalizations (95% CI = 2.20, 197.60; *p* = 0.021). The adjusted unemployment odds for people with psychiatric hospitalizations are 12.85 as compared to people without psychiatric hospitalizations (95% CI = 2.5, 235.6; *p* = 0.015). Despite these findings, no significant association was found between psychiatric hospitalization history MGM and injury severity (see Table 9).

**Table 9:** Unadjusted and adjusted logistic regression models predicting injury severity, discharge location, employment, and PTA days outcomes for Psychiatric Hospitalization Marginalized Group Membership (N = 254).

**Variable**	**Unadjusted**			**Adjusted**		
	**OR (SD)**	**95% CI**	***p*-value**	**OR (SD)**	**95% CI**	***p*-value**
Injury severity—severe^a^	0.94 (1.49)	(0.42, 2.04)	0.873	0.87 (1.53)	(0.37, 1.98)	0.735
Discharge location—to community	0.39 (1.44)	(0.18, 0.79)	0.010*	0.38 (1.47)	(0.17, 0.80)	0.011*
Employment—unemployed	10.91 (2.81)	(2.20, 197.60)	0.021*	12.85 (2.84)	(2.50, 235.60)	0.015*
	**Difference (SD)**	**95% CI**	***p*** **-value**	**Difference (SD)**	**95% CI**	***p*** **-value**
PTA days	10.51 (4.23)	(2.20, 18.90)	0.014*	10.84 (4.30)	(2.40, 19.30)	0.013*

Adjusted for age, sex, and cause of TBI. Sample size may vary due to missing data. The reference group for all odds ratios are participants who reported no psychiatric hospitalizations.

^†^*p* < 0.10, *p < 0.05, **p < 0.01, ***p < 0.001.

^*a*^Reference level of Injury Severity: complicated mild or moderate injury.

### Psychiatric disorders MGM

As described in Table 10, people with psychiatric disorders had 0.41 times the odds, or 59% lower odds, of being discharged to a community setting as compared to people without psychiatric disorders (95% CI = 0.23, 0.71; *p* = 0.002). After adjusting for age, sex, and severity of TBI, people with psychiatric disorders had 0.45 times the odds, or 55% lower odds, of being discharged to a community setting as compared to people without psychiatric disorders (95% CI = 0.25, 0.81; *p* = 0.008). No significant associations were found between injury severity, PTA days, and psychiatric disorders. Despite previous findings with respect to people with psychiatric hospitalizations and employment outcomes, for people with psychiatric hospitalization, no significant differences were found in unadjusted (*p* = 0.142) or adjusted models (*p* = 0.120).

**Table 10:** Unadjusted and adjusted regression models predicting injury severity, discharge location, employment, and PTA days outcomes for Psychiatric Disorders Marginalized Group Membership (N = 254).

**Variable**	**Unadjusted**			**Adjusted**		
	**OR (SD)**	**95% CI**	***p*-value**	**OR (SD)**	**95% CI**	***p*-value**
Injury severity—severe^a^	0.96 (1.35)	(0.53, 1.72)	0.892	0.90 (1.39)	(0.47, 1.71)	0.759
Discharge location—to community	0.41 (1.33)	(0.23, 0.71)	0.002**	0.45 (1.35)	(0.25, 0.81)	0.008**
Employment—unemployed	1.80 (1.49)	(0.85, 4.11)	0.142	1.92 (1.52)	(0.87, 4.57)	0.120
	**Difference (SD)**	**95% CI**	***p*** **-value**	**Difference (SD)**	**95% CI**	***p*** **-value**
PTA days	1.31 (3.41)	(−5.40, 8.00)	0.701	2.15 (3.58)	(−4.90, 9.20)	0.549

Adjusted for age, sex, and cause of TBI. Sample size may vary due to missing data. The reference group for all odds ratios are participants who reported no psychiatric disorders.

^†^*p* < 0.10, *p < 0.05, **p < 0.01, ***p < 0.001.

^*a*^Reference level of Injury Severity: complicated mild or moderate injury.

### Incarceration history MGM

Among 155 participants (see Table 11), individuals with incarceration history had 8.15 (95% CI = −1.2, 17.5) more PTA days than people without a history of incarceration, but this association was not significant (*p* = 0.087). After adjusting for age, sex, and cause of TBI, formerly incarcerated individuals had 6.05 more PTA days, but this association was not significant (95% CI = −4.1, 16.2; *p* = 0.24).

**Table 11:** Unadjusted and adjusted regression models predicting injury severity, discharge location, employment, and PTA days outcomes for incarceration history marginalized group membership (N = 155).

**Variables**	**Unadjusted**			**Adjusted**		
	**OR (SD)**	**95% CI**	***p*-value**	**OR (SD)**	**95% CI**	***p*-value**
Injury severity—severe^a^	1.46 (1.44)	(0.71, 2.99)	0.299	1.23 (1.49)	(0.56, 2.70)	0.604
Discharge location—to community	0.42 (1.42)	(0.21, 0.84)	0.014*	0.32 (1.49)	(0.14, 0.70)	0.005**
Employment—unemployed	2.20 (1.72)	(0.81, 7.09)	0.146	2.59 (1.80)	(0.87, 9.02)	0.105
	**Difference (SD)**	**95% CI**	***p*** **-value**	**Difference (SD)**	**95% CI**	***p*** **-value**
PTA days	8.15 (4.72)	(−1.2, 17.5)	0.087^†^	6.05 (5.12)	(−4.10, 16.20)	0.240

Adjusted for age, sex, and cause of TBI. Sample size may vary due to missing data. The reference group for all odds ratios are participants who reported no incarceration history.

^†^*p* < 0.10, *p < 0.05, **p < 0.01, ***p < 0.001.

^*a*^Reference level of injury severity: complicated mild or moderate injury.

Individuals with incarceration history had 0.42 times the odds, or 58% lower odds, of being discharged to the community than people without incarceration history (95% CI = 0.21, 0.84; *p* = 0.014). After adjusting for age, sex, and the cause of TBI, people with incarceration history had 0.32 times the odds, or 68% lower odds, of being discharged to a community setting as compared to people without incarceration history (95% CI = 0.14, 0.70; *p* = 0.005). No significant association was found between incarceration history and TBI severity, PTA days, or employment status.

## Discussion

Our research findings revealed that high marginalization has a compounding negative effect across TBI severity, recovery, and employment outcomes; however, when assessing MGMs in isolation, certain associations were not significant in unadjusted or adjusted models. Discharge location had mixed findings and was not significantly associated to those with high MGM. Increased marginalization, regardless of the combination, is negatively associated with key TBI outcomes. These results demonstrate that the burden of multiple MGMs, in addition to the chronic effects of TBI, pose significant challenges. Our analyses on the intersection of individual's marginalization further expand previous research findings documenting increased risk, attributed to systemic vulnerabilities, for injury severity, delayed care, longer treatment, and worse recovery (7, 8). They also provide new insights on key outcomes related to discharge location and employment.

Injury severity was measured according to GCS scores—a standard measure of injury severity in acute settings that is used to inform decisions about treatment and resource allocation (41). Low GCS scores, denoting a more severe injury, have been associated with high mortality rates and poorer prognosis (37). TBI patients with high MGM revealed a significantly higher likelihood to sustain severe TBIs, yet only two of the studied MGMs showed significant associations of sustaining severe TBI when studied separately. Individuals with TBI identifying racial and ethnic minority status MGM, in particular Blacks and Hispanics, and less than HS education, MGM presented increased odds of sustaining severe TBI as opposed to complicated mild-moderate injury. Individuals with HS education and college education or above were at significantly decreased odds of sustaining a severe TBI when compared to individuals with less than HS education. Our findings are also consistent with previous studies documenting that racial and ethnic minorities are more likely to sustain severe TBIs, including those with high systemic vulnerabilities (7, 42). These findings demonstrate a need for targeted TBI prevention efforts for those in marginalized communities that account for health literacy and cultural responsiveness.

Recovery was characterized in terms of average PTA days, which is considered a good predictor of long-term TBI recovery outcomes (38). PTA duration, when compared to GCS or loss of consciousness, is also a stronger predictor of functional and cognitive recovery and return to employment (38, 43). This study found that TBI patients with high MGM are significantly more likely to experience longer PTA, which is consistent with findings by Fuentes et al. (7). When assessing MGMs individually, participants with racial and ethnic minority status, LEP, and psychiatric hospitalization are associated with having significantly longer PTA. Further exploration within the racial and ethnic minority status and LEP MGMs showed that longer PTA days are associated with participants who were identified as Black, Hispanic, and spoke a language other than English or Spanish. These findings underscore the critical importance of addressing multidimensional factors concurrent with TBI recovery, as the long-term effects of TBI can place an additional burden on individuals and their economic stability. Multidisciplinary approaches accounting for structural inequities are required to meet the healthcare needs of marginalized TBI patients across the recovery.

Community integration is a key rehabilitation goal after TBI, with appropriate discharge location considered an important factor to optimize recovery (44). Although discharge to a community setting is an optimal outcome, individuals may benefit from receiving further care and support for any lasting injury effects (45). Findings reveal no significant difference between discharge location and individuals' level of marginalization. Previous studies have linked greater injury severity, functional status, and longer acute care stay with a decreased likelihood of being discharged to the community (44), while those who are uninsured are an increased likelihood of being discharged home (46). Upon closer observation, results within MGM groups were mixed. Discharge to a facility is associated with psychiatric hospitalizations, psychiatric disorders, and incarceration history MGM. On the other hand, discharge to the community is associated with participants who have LEP, in particular, those who speak Spanish. A possible explanation may be due to insurance status (47), in our sample, about one-third relied on financial aid from the hospitals and two-fifths relied on Medicaid. Sociodemographic factors have been studied in relation to community integration and discharge location after TBI (44, 48, 49); these findings support the importance of accounting for concurrent marginalization at the time of injury to ensure appropriate care delivery and long-term management.

Employment after TBI is impacted for all survivors, with sociodemographic factors, injury severity, and disability levels pre- and post-injury playing a paramount role in return to work (30, 50). Besides economic toll and loss of benefits, employment is an important psychosocial predictor of wellbeing, quality of life, recovery, and participation after TBI (30, 51, 52). Given the multidimensional importance of employment, our study sought to identify differences between marginalized groups and employment outcomes 1 year-post injury. TBI patients with high MGM had significantly reduced competitive paid employment 1 year after injury. When studied separately, unemployment 1-year post-injury is associated with less than HS education, homelessness, and psychiatric hospitalization. Upon closer review, individuals with college-level education or above were significantly at decreased odds of being unemployed when compared to individuals with less than HS education. These findings align with previous studies documenting higher education levels with an increased chance of workforce re-entry (53), as education is a key independent predictor of long-term functional and neurocognitive outcomes (54). Our findings linking increased unemployment to homelessness and psychiatric hospitalization are particularly concerning, as TBI is associated with increasing the factors that lead to homelessness and the development or worsening of psychological symptoms and disorders (15, 55). The study also revealed that Blacks and Hispanics had significantly higher odds of unemployment when compared to white participants, which coincides with previous research (56). TBI effects cause a large financial toll on the individual and their social network as they manage the cost of care, while they experience difficulty finding work or working at their previous capacity. Given the impact marginalization has on employment outcomes, there is a need for service programs to connect marginalized community members to effective vocational rehabilitation services considering their concurrent needs.

This study's findings reveal that individual MGMs had varying effects, yet despite these singular differences when four or more marginalization factors intersect, there is a compounding negative effect across TBI severity, recovery, and employment outcomes. Currently, research, seeking an understanding of TBI outcomes and recovery, fails to assess the intersection of marginalization factors and frequently focuses on a singular dimension. It is important to identify individuals most at risk of poor outcomes in their TBI recovery, as well as explanations for their heightened risk. Previous research has documented health disparities, yet causal mechanisms remain unclear as individuals' social determinants of health, structural inequalities, and racism intersect. Therefore, economic, social, and structural disparities, along with unequal access to psychosocial services and clinical care, can have detrimental effects on TBI outcomes. Our approach to TBI can no longer remain one-dimensional. Instead, we must take a holistic, translational research, an interdisciplinary approach to comprehensively understand the real-world experience of living with TBI and identify evidence-based strategies to improve health outcomes. By better understanding the interaction between TBI and where the person is positioned in their community, robust, comprehensive models can be developed to identify effective population-based prevention and treatment strategies, healthcare access and allocation, and policies (33).

### Limitations

The results of this study do not indicate causality as MGM does not explain poor health outcomes. Rather these marginalization factors point to social and structural inequities enmeshed within society that impact TBI recovery and require further exploration. The generalization of the findings to rural and suburban communities may also be limited as recruitment took place at a TBIMS center in a densely, urban area in the US. Our experimental design and the performed secondary analysis limited the availability of variables related to social inequalities. It is unclear if individuals that reported marginalized group membership also experienced social inequalities such as racism or discrimination. Due to the different statistical tests performed, we used a *p*-value to compare the results as opposed to confidence interval and effect. We did observe that our confidence intervals widened as our effect sizes increased. Despite exploring within MGM groups, such as racial and ethnic minorities, LEP, and education using multiple categories, the analysis for other MGM groups remained dichotomous, making it difficult to discern the nuances within each marginalized group and intersections. Contrary to findings reported in the literature, no significant associations were found for Asian individuals with TBI (probably due to a limited sample size) nor in persons reporting substance abuse. Due to the longitudinal nature and the marginalized communities studied, some participants were lost to follow-up after discharge from acute rehabilitation care. Furthermore, given that we were exploring employment outcomes, our sample excluded elderly populations above the age of 66 years. Participants in this study received inpatient rehabilitation at two comprehensive rehabilitation centers in New York City, which may not be representative of the care access available to other marginalized community members in the acute and long-term setting.

### Future directions

This study focuses on the period from the time of injury to 1-year post-injury; however, the chronic nature of TBI, with health effects presenting even years after the injury, demands longitudinal assessments across the recovery. Mixed-method and community-based participatory research approaches are necessary to better characterize the representation of community perspectives on managing TBI and marginalization and the root causes of health disparities. Multi-level modeling is essential to comprehensively assess the role of marginalized identities, structural barriers, and historical processes that link to systems of oppression and power in influencing health outcomes. Further studies with robust correction and multi-outcome analysis are warranted. Future research is needed with a larger sample of TBI patients from historically disadvantaged and marginalized groups, as well as community-based samples that may or may not have received inpatient rehabilitation or follow-up care.

## Conclusion

This study provides key insights on how marginalization cumulatively and differentially interacts with TBI outcomes and recovery, from the time of injury to 1-year post-injury. Our research revealed a cumulative negative effect with high marginalization across TBI injury severity, recovery, and employment outcomes; however, the effect differs across the eight MGMs and four outcomes. With prevailing concerns about individuals with TBI being disproportionately represented across marginalized groups, for instance, individuals who have been in the criminal justice system or who have unstable housing (55, 57), there is a need for research to understand the interaction between TBI and existing or new marginalization. The contribution of these results and methodology present insights on the impact of marginalization within an already marginalized group, or those who sustain TBI, illustrating disparities and how they intersect. Our focus on an intersectional, urban-based TBI population highlight key areas for clinical and research-practice recommendations.

To disrupt the cycle of marginalization and its multilevel negative impact, it is important to address the multidimensional factors concurrent to TBI recovery by increasing and improving interdisciplinary care and service access and provision, and research initiatives across TBI recovery. Currently, a lack of consensus exists on the types and needs of long-term service delivery after TBI (58); therefore, interdisciplinary approaches are necessary to better adapt and test promising rehabilitation programs and therapies, such as vocational rehabilitation services, to the needs of individuals from marginalized communities. Acute rehabilitation centers should seek to develop partnerships with community-based organizations to support individuals with TBI to make a transition back to the community, as well as to connect individuals to resources, social support, and services. Finally, further research is warranted that will contribute to the development of prevention, clinical, and community integration services and programs, practices, and policies that advance health equity among marginalized groups with TBI.

## Data availability statement

The original contributions presented in the study are included in the article/supplementary materials, further inquiries can be directed to the corresponding author.

## Ethics statement

The studies involving human participants were reviewed and approved by Institutional Review Boards at Grossman School of Medicine, NYU Langone Health and Bellevue Hospital, NYC Health and Hospitals. The patients/participants provided their written informed consent to participate in this study.

## Author contributions

All authors listed have made a substantial, direct, and intellectual contribution to the work and approved it for publication.

## Funding

The National Institute on Disability Independent Living and Rehabilitation Research (NIDILRR) within the Administration for Community Living (ACL) 90DP0047 and 90DPTB0100 grants funded this research.

## Conflict of interest

The authors declare that the research was conducted in the absence of any commercial or financial relationships that could be construed as a potential conflict of interest.

## Publisher's note

All claims expressed in this article are solely those of the authors and do not necessarily represent those of their affiliated organizations, or those of the publisher, the editors and the reviewers. Any product that may be evaluated in this article, or claim that may be made by its manufacturer, is not guaranteed or endorsed by the publisher.

## Author disclaimer

The contents of this article are solely the author's responsibility and are not representative of the views of the awarding agencies nor endorsement by the Federal Government.

## Footnotes

^1^For further details about the TBIMS National Database can be found at: www.tbindsc.org.
